# The Germination Paradox in Sorghum: A Review

**DOI:** 10.3390/foods15030569

**Published:** 2026-02-05

**Authors:** Yogita Sharma, Nidhish Francis, Christopher Blanchard, Abishek Bommannan Santhakumar

**Affiliations:** School of Dentistry and Medical Sciences, Faculty of Science and Health, Charles Sturt University, Wagga, NSW 2650, Australia; ysharma@csu.edu.au (Y.S.); nfrancis@csu.edu.au (N.F.); cblanchard@csu.edu.au (C.B.)

**Keywords:** *Sorghum bicolor*, germination, polyphenols, anti-nutrients, bioaccessibility, bioavailability, bioactivity, functional foods

## Abstract

Sorghum (*Sorghum bicolor* L. Moench) is a climate-resilient cereal with significant potential as a functional food due to its distinctive polyphenolic profile, including rare 3-deoxyanthocyanidins (3-DXAs). Broader utilisation of sorghum is limited by low protein digestibility and the presence of anti-nutritional factors, such as condensed tannins and phytates. This review consolidates current evidence on germination as a bioprocessing strategy to address these limitations and enhance the bioactivity of sorghum polyphenols. Germination activates endogenous hydrolytic enzymes, such as phytases and esterases, and upregulates the phenylpropanoid pathway through phenylalanine ammonia-lyase, which promotes the release of cell wall-bound phenolic acids and the de novo synthesis of flavonoids. A “germination paradox” is identified, in which qualitative shifts toward lower-molecular-weight, more bioaccessible aglycones enhance antioxidant and anti-inflammatory efficacy, even when total phenolic content fluctuates. The review also examines the effects of germination on digestive release, transepithelial transport, and colonic microbial transformation of phenolics. Finally, genotype- and process-dependent optimisation windows, typically 48–72 h, are delineated to balance anti-nutrient reduction with phytochemical retention, providing a basis for the development of germinated sorghum-based functional foods and nutraceuticals.

## 1. Introduction

Sorghum (*Sorghum bicolor* L. Moench) is among the most ancient and agronomically resilient cereal crops, ranking fifth in global cereal grain production after maize, wheat, rice, and barley [1]. Its cultivation is concentrated in semi-arid regions of Africa and Asia, where it serves as a primary dietary staple for nearly half a billion people [2]. The crop’s inherent drought tolerance, heat resistance, and ability to thrive in marginal soils underscore its significance as a climate-adaptive cereal vital for global food security amid increasing population pressures and climatic variability [3,4,5]. In addition to its traditional roles as a subsistence grain and livestock feed, sorghum has gained prominence in industrialised markets as a naturally gluten-free whole grain, stimulating research into its nutritional composition, bioactive constituents, and functional applications in human nutrition [6,7]. Sorghum is notable for its rich and chemically diverse polyphenol content [8,9]. Pigmented varieties, in particular, accumulate significant levels of phenolic acids, flavonoids, condensed tannins, and the unique 3-DXAs within the grain pericarp and outer endosperm [2,8]. These compounds impart characteristic pigmentation and exhibit pronounced antioxidant and anti-inflammatory activities, including reactive oxygen species scavenging and modulation of inflammatory signalling pathways, which are associated with reduced risk of chronic diseases such as cardiovascular disease (CVD), type 2 diabetes mellitus (T2DM), and certain cancers [10,11]. The diversity and concentration of sorghum polyphenols are strongly determined by grain pigmentation and genotype, influencing both sensory properties and physiological bioefficacy [8,10].

However, the nutritional and functional exploitation of sorghum is constrained by several intrinsic compositional and anatomical aspects. Anti-nutritional constituents such as phytate (inositol hexaphosphate) and condensed tannins, especially in pigmented cultivars, can chelate divalent minerals and form insoluble protein–tannin complexes, consequently lowering nutrient bioavailability [12]. Moreover, the compact organisation of starch granules and prolamin (kafirin) protein bodies within the endosperm matrix imposes spatial constraints on enzymatic hydrolysis, resulting in comparatively reduced starch and protein digestibility relative to other cereals [13]. These structural and chemical features also limit the release and gastrointestinal accessibility of polyphenolic compounds embedded in the grain matrix, causing diminished metabolic utilisation of sorghum in its native or minimally processed form [8,14,15].

To surmount these obstacles and more fully realise sorghum’s potential as a health-promoting ingredient, a variety of processing and bioprocessing strategies have been applied, including decortication and milling, thermal treatments, extrusion, fermentation and germination. Such processes modify grain microstructure, reduce anti-nutrient levels and alter the profile and extractability of phytochemicals, thus affecting both nutrient digestibility and bioactive functionality [15,16]. Among these approaches, germination (sprouting) functions as a central bioprocess. Germination is a biologically driven modification initiated by controlled hydration and incubation, which activates endogenous hydrolytic and biosynthetic enzyme systems, remodels storage macromolecules and can qualitatively and quantitatively transform the sorghum polyphenol profile [17]. Emerging evidence indicates that germination-induced changes are accompanied by increases in antioxidant capacity, modulation of inflammatory responses and improvements in mineral and polyphenol bioaccessibility [13,15,18].

Although research on germinated sorghum is expanding, the literature remains fragmented due to variations in genotypes, germination protocols, and analytical methodologies. Many studies focus primarily on total phenolic content or general antioxidant assays, often without integrating compositional analysis, bioaccessibility, or downstream biological activity. This fragmentation hinders a comprehensive understanding of how germination alters sorghum polyphenols, the translation of these changes into antioxidant and anti-inflammatory effects, and the extent to which germination enhances the bioaccessibility and bioavailability of sorghum-derived polyphenols in physiologically relevant contexts. This review aims to elucidate the mechanisms by which germination remodels sorghum polyphenols, enhances their antioxidant and anti-inflammatory activities, and improves their bioaccessibility and bioavailability, with particular attention to the development of germinated sorghum-based functional foods.

This review assesses germination as a bioprocessing strategy to enhance the functional value of sorghum polyphenols. Section 2 contextualises germination within the broader biochemical and technological landscape of cereal and legume processing. Section 3 details the polyphenolic composition of sorghum, including major phenolic classes, anatomical distribution, and genotype–environment interactions, to establish a compositional baseline. Section 4 explores germination-induced modifications of sorghum polyphenols, emphasising changes in phenolic acids, flavonoids, and condensed tannins, and their impact on antioxidant and anti-inflammatory properties. Section 5 addresses the influence of germination on polyphenol bioaccessibility and bioavailability, covering digestive release, absorption, metabolism, and comparisons with other cereals and legumes. Section 6 synthesises evidence from in vitro, in vivo, and human studies regarding biological activity. The review concludes by identifying key methodological gaps and future research priorities to advance functional food and nutraceutical applications.

## 2. Germination in Cereals and Legumes

### 2.1. General Biochemistry of Germination

**Figure 1:**
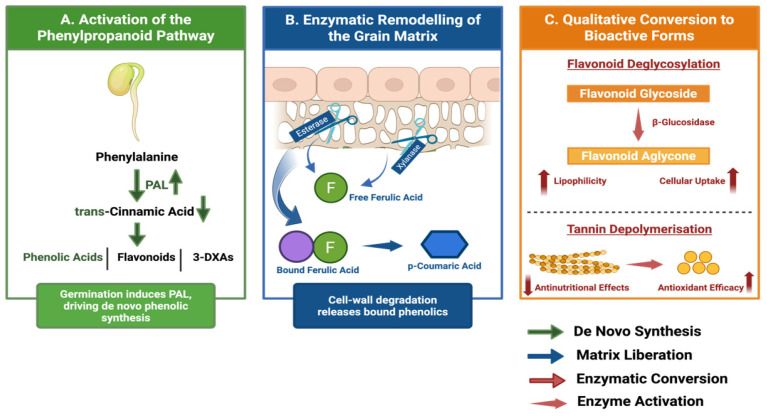
Germination induces biosynthesis, liberates matrix-bound phenolics, and converts polymeric compounds into low-molecular-weight (LMW) bioactive forms.

Germination constitutes a pivotal developmental transition in which the seed shifts from a quiescent, storage-centric state to an actively metabolising organism [19]. Upon imbibition, water uptake triggers respiratory reactivation and the mobilisation of storage macromolecules to support embryonic growth, a process orchestrated by the sequential activation of endogenous hydrolytic enzymes, principally amylases, proteases and phytases, which hydrolyse complex reserves into simpler, bioaccessible nutrients [15]. Biochemically, this remodelling proceeds via two complementary mechanisms: the enzymatic degradation of anti-nutritional factors and the structural modification of the seed matrix. Upregulation of phytase activity hydrolyses phytic acid (inositol hexakisphosphate), releasing divalent minerals, such as iron, zinc and calcium, previously sequestered by phytic complexes and consequently boosting mineral bioavailability [20,21]. Concurrent proteolytic and amylolytic activities degrade storage proteins and depolymerise starch granules, increasing protein and starch digestibility [15]. Importantly, germination also entails biosynthetic activation: the induction of phenylalanine ammonia-lyase (PAL), the rate-limiting enzyme of the phenylpropanoid pathway, drives de novo synthesis of phenolic compounds and the accumulation of free phenolic acids and flavonoids [22]. Simultaneously, enzymatic hydrolysis of cell wall constituents such as arabinoxylans liberates phenolics previously bound within the matrix, altering their solubility and potential gastrointestinal accessibility [15] (Figure 1).

### 2.2. Evidence from Other Cereals and Pulses

Empirical data across cereals and legumes demonstrate that germination exerts bioenhancing effects (refer Table 1), although the magnitude and specific outcomes depend on genotype and process parameters [15,23]. In pulses such as chickpea, mung bean and lentil, 24–48 h germination regimes have been reported to reduce phytic acid by 30–75%, changes that correlate with improved mineral availability; these pulses also frequently exhibit increased total phenolics and antioxidant activity, with documented elevations in polyphenols and tocopherols following germination [20,24,25,26]. In cereal species, including barley and corn, germination markedly alters macronutrient composition, reducing lipid and protein fractions, while increasing radical scavenging activity as measured by assays such as DPPH, indicative of a shift towards a relatively antioxidant-enriched profile despite consumption of stored energy reserves [15,22]. Millet studies resemble these trends and illustrate clear time-dependent anti-nutrient degradation; for example, phytate concentrations decreased progressively from 0.836 g/100 g to 0.173 g/100 g after 72 h of germination [27]. Collectively, these observations position germination as an effective processing technique to enhance nutrient and phytochemical accessibility across several seed types.

### 2.3. Germination as a Bioenhancing Strategy

Taken together, the biochemical and empirical evidence frames germination as an effective, natural bioenhancing strategy that dismantles the rigid seed matrix and reduces anti-nutritional barriers to unlock otherwise inaccessible nutrients and functional phytochemicals [15] (refer to Table 1). Although the underlying mechanisms of cell wall hydrolysis, phytate breakdown and activation of biosynthetic pathways are broadly conserved among seeds, their practical application to *Sorghum bicolor* is of particular interest because of sorghum’s abundant, matrix-entrapped polyphenolic pool. Consequently, germination provides a targeted approach to mobilise these bioactive compounds, but its efficacy will depend on grain-specific properties and processing parameters, underscoring the need for genotype and context-aware protocols [41].

## 3. Sorghum Polyphenols

### 3.1. Chemical Classes

The phytochemical complexity of *Sorghum bicolor* arises from a broad spectrum of phenolic metabolites, secondary plant constituents characterised by one or more hydroxyl groups attached to aromatic rings [42]. These phenolic entities are principally categorised into phenolic acids, flavonoids, and condensed tannins, whose concentration and qualitative distribution are profoundly influenced by the genetic lineage and pigmentation of the sorghum cultivar [10,43,44]. Phenolic acids occur predominantly in conjugated forms, esterified to cell wall polysaccharides and lignin components, thus influencing both their extractability and bioavailability [45].

Within this class, hydroxybenzoic and hydroxycinnamic acids constitute the two major subgroups, the latter being quantitatively superior in most sorghum genotypes [46,47]. Among these, ferulic acid predominates as the principal phenolic acid, followed by *p*-coumaric and caffeic acids [48]. These hydroxycinnamate derivatives are potent radical scavengers, functioning as chain-breaking antioxidants that stabilise reactive oxygen and nitrogen species through resonance delocalization of phenoxyl radicals. Their association with cell wall polymers, however, restricts their in vivo release and intestinal absorption, underscoring the need for processing-induced de-esterification reactions to enhance their physiological efficacy.

Flavonoids, another prominent group of polyphenols, share a conserved C_6_–C_3_–C_6_ carbon skeleton composed of two aromatic rings joined by a heterocyclic pyran ring. Sorghum accumulates several flavonoid subclasses, particularly flavones such as luteolin and apigenin, and flavanones including naringenin and eriodictyol, which are especially abundant in yellow pericarp varieties [42,49]. These compounds demonstrate pronounced antioxidative and anti-inflammatory activities, attributed to their capacity to modulate redox-sensitive transcription factors and inhibit pro-inflammatory cytokine release [50,51]. Their pleiotropic biological functions, ranging from endothelial protection to neurovascular regulation, have established flavonoids as integral contributors to the health-promoting profile of sorghum [10].

Condensed tannins, or proanthocyanidins, are polymeric flavan-3-ol aggregates primarily composed of catechin and epicatechin subunits [52]. Their synthesis is genotype-specific, occurring predominantly in “tannin” or brown sorghum lines where they confer astringency and dark pigmentation to the grain [42]. While these high-molecular-weight polyphenols exhibit strong protein and mineral-binding capacities that may impair nutrient absorption, emerging evidence redefines them as potent antioxidants and metal chelators, with the ability to modulate lipid oxidation and inflammatory signalling [10]. This duality, anti-nutritional yet bioactive, positions condensed tannins as functional compounds whose health relevance depends on their structural degree of polymerization and dietary context.

### 3.2. The Unique 3-Deoxyanthocyanidins (3-DXA) of Sorghum

A distinctive hallmark of sorghum’s polyphenolic composition is the accumulation of 3-DXA, a rare subclass of anthocyanin analogues scarcely encountered in other cereal or horticultural crops [49]. Structurally, these pigments are characterised by the absence of a hydroxyl group at the C3 position of the heterocyclic C-ring, a modification that imparts exceptional stability against thermal, photolytic, and pH-induced degradation compared to conventional 3-hydroxylated anthocyanins [49]. This enhanced structural rigidity and reduced polarity confer not only greater pigment persistence during processing but also increased suitability for industrial application as natural food colourants and functional nutraceuticals.

The predominant 3-DXA compounds in sorghum include luteolinidin and apigeninidin, together with their O-methylated derivatives, 5-methoxyluteolinidin and 7-methoxyapigeninidin [49,53]. These molecules are synthesised via the flavonoid biosynthetic pathway and are often induced as phytoalexins under biotic stress conditions, such as fungal invasion, or abiotic oxidative stress [54]. The accumulation of 3-DXA is genotype-dependent, reaching particularly high concentrations in black or red pericarp sorghums, where bran fractions may contain up to 16 mg/g of pigment levels, surpassing those of typical anthocyanin-rich fruits such as blueberries or blackcurrants [10].

Beyond their pigmentary and technological attributes, 3-DXA demonstrates pronounced bioactivity in mammalian systems. Experimental models have revealed that luteolinidin and apigeninidin exert strong cytotoxic and antiproliferative effects against diverse human cancer cell lines, effects often exceeding those of their hydroxylated analogues [11,54]. Mechanistically, these compounds are believed to modulate redox homeostasis and apoptosis signalling by generating controlled oxidative stress and inhibiting pro-oncogenic transcriptional pathways. Thus, the 3-DXA group not only defines sorghum’s unique chromatic identity but further accentuates its emerging nutraceutical potential as a source of chemically stable and biologically potent phenolic pigments.

### 3.3. Anatomical Distribution

The health-promoting attributes of *Sorghum bicolor* are closely determined by the anatomical localization of its phenolic constituents within the grain architecture. Unlike macronutrient fractions, which are concentrated within the starchy endosperm, the majority of phenolic compounds estimated to exceed 90% of total content are confined to the peripheral tissues, including the pericarp, aleurone layer, and, where genetically expressed, the pigmented testa [10]. The pericarp, constituting the outermost bran fraction, represents the principal reservoir for phenolic acids and, in coloured genotypes, for the distinctive 3-DXAs [55,56,57]. Immediately beneath the pericarp lies the testa, a phenol-rich layer that develops only in varieties possessing dominant *B1* and *B2* alleles; it serves as the exclusive site for the biosynthesis and accumulation of condensed tannins [52]. In contrast, the endosperm, which constitutes the bulk of the grain mass, exhibits minimal phenolic enrichment and is largely composed of starch granules embedded within a hydrophobic protein matrix [10].

This spatial compartmentalization of polyphenols has direct implications for post-harvest processing and the resultant nutritional value of sorghum-based products. Mechanical debranning procedures, such as decortication, employed to produce refined flours with improved palatability and reduced fibre content, often remove these phenolic-dense outer layers, thereby depleting the antioxidant and anti-inflammatory potential of the final product [10]. Consequently, preserving the bran fraction through whole grain utilisation is imperative to maintain the bioactive integrity of sorghum and to maximise its potential contribution to dietary health promotion.

### 3.4. Free, Conjugated and Bound Phenolics

Phenolic compounds in the sorghum kernel exist in three operationally defined fractions, free, conjugated, and bound, whose proportions critically determine their extraction efficiency, gastrointestinal release, and systemic bioavailability [45]. Free phenolics are solvent-extractable, low-molecular-weight entities, typically located in the vacuolar compartments, and comprise monomeric or oligomeric forms that are directly available for absorption in the upper GI tract (gastrointestinal tract). Conjugated phenolics, although soluble, are covalently linked to small molecules such as sugars or organic acids via ester or glycosidic bonds, thereby influencing their polarity and enzymatic hydrolysis during digestion [45].

In contrast, the insoluble-bound phenolic fraction, quantitatively dominant in cereal, is covalently integrated within the plant cell wall architecture, forming ester and ether linkages with polysaccharides (notably arabinoxylans), lignin, and structural proteins [45,58]. Liberation of these bound phenolics requires chemical or enzymatic hydrolysis to disrupt the structural macromolecular matrix [45,59,60]. This fraction is of particular physiological interest as it resists upper intestinal digestion and becomes available for colonic fermentation, where microbial metabolism can generate bioactive phenolic metabolites capable of exerting systemic antioxidant and anti-inflammatory effects.

The proportional distribution of these phenolic forms is highly genotype-dependent. In black and brown pericarp sorghums, up to 80% of total flavonoids occur in the free fraction, suggesting superior initial bioaccessibility [10]. Conversely, white and red genotypes exhibit markedly lower free fractions, with a greater proportion of phenolics in bound forms. For phenolic acids, particularly ferulic acid, the bound state predominates across virtually all cereal matrices, reflecting its extensive cross-linking with cell wall arabinoxylans [45]. The metabolic fate of each fraction therefore differs fundamentally: free and conjugated forms may undergo rapid intestinal absorption, whereas bound phenolics largely reach the colon, serving as substrates for the gut microbiota and contributing to the generation of phenolic-derived postbiotics. Thus, the ratio of free to bound phenolics represents a biochemical determinant of both immediate antioxidant potential and longer-term microbial-mediated health benefits.

### 3.5. Influence of Genotype and Environment on Phenolic Profiles

The phenolic composition of sorghum is not a fixed attribute, but a dynamic phenotype shaped by a complex interplay between environmental stimuli and genetic determinants. At the genomic level, the qualitative pattern of phenolic biosynthesis is primarily determined by the expression of structural and regulatory genes that control the flavonoid and phenylpropanoid pathways [10,61,62]. The pigmentation and polyphenolic composition of the pericarp and testa are defined by specific allelic combinations: dominant *R* and *Y* alleles confer red pigmentation essential for 3-deoxyanthocyanidin biosynthesis, while dominant *B*1 and *B*2 alleles are required for testa formation and subsequent tannin deposition [42,49,63,64]. The *S* (spreader) gene further modulates tannin distribution by facilitating diffusion of these polymers from the testa into the pericarp, enhancing the total phenolic content of the grain [42,65]

Environmental parameters exert substantial quantitative modulation on these genetically defined pathways. Abiotic stresses such as elevated temperature, drought and ultraviolet radiation act as potent inducers of the phenylpropanoid metabolic cascade, leading to increased accumulation of defensive phenolic metabolites that mitigate oxidative and photochemical damage [66,67]. Comparative analyses across cereal species indicate that environmental factors, including geographical location, soil composition, and annual climatic fluctuations, can, in certain instances, exert a greater influence on total phenolic content than genotype alone [42,56,68]. This genotype-by-environment (G × E) interaction implies that identical sorghum genotypes cultivated under divergent ecological conditions may display considerable heterogeneity in their polyphenolic profiles, with direct implications for the consistency of functional food formulations and nutraceutical applications.

Reliance on total phenolic content (TPC) as a unidimensional index is, therefore, methodologically insufficient to characterise such complex biochemical variation. The Folin–Ciocalteu assay, though widely applied, lacks specificity and fails to distinguish between structurally distinct phenolic subclasses, conjugation states, and degrees of polymerization that ultimately dictate biological activity [46,69,70]. Consequently, comprehensive chromatographic and spectrometric approaches, such as liquid chromatography mass spectrometry (LC–MS) and high-performance liquid chromatography (HPLC) are indispensable for elucidating the compositional fingerprint of sorghum phenolics [46,48]. Such analytical precision is essential not only for genotype selection in breeding programmes but also for ensuring reproducible bioefficacy in sorghum-derived functional foods and therapeutic formulations.

## 4. Effects of Germination

### 4.1. Metabolic Activation and Phytochemical Remodelling

Within the broader discourse of cereal science and functional food development, germination, often referred to as “malting” in industrial contexts, represents far more than a simple physiological transition from seed dormancy to vegetative growth. Rather, it constitutes a profound metabolic awakening of the sorghum grain (*Sorghum bicolor* L. Moench), initiating a cascade of tightly regulated biochemical events that fundamentally restructure the seed’s phytochemical landscape [9,71,72,73]. From the perspectives of food science, nutrition and agronomy, germination provides a naturally derived and cost-effective strategy to valorise sorghum, transforming it from a coarse, often underutilised, subsistence grain into a functional ingredient characterised by enhanced bioactive profiles and improved nutritional efficacy.

The central premise underpinning this section is that germination functions as a form of “nature’s refining step”. Conventional processing techniques such as thermal treatments (roasting, extrusion) or mechanical fractionation (decortication, milling) frequently degrade heat-sensitive bioactives or physically remove the bran layers in which the majority of sorghum polyphenols are concentrated [8,74]. In contrast, germination exploits endogenous enzymatic systems to synthesise, liberate and potentiate phenolic compounds through a dual mechanism involving the hydrolytic release of cell wall-bound phenolics via esterases and glycosidases, alongside de novo synthesis of phenolic acids and flavonoids through upregulation of the phenylpropanoid pathway [72,75,76].

Elucidating these transformations requires detailed consideration of the interplay between enzymatic activity, particularly phenylalanine ammonia-lyase (PAL) and feruloyl esterases, the complex structural chemistry of sorghum polyphenols, including condensed tannins and the distinctive 3-DXAs, and external processing variables such as germination duration, moisture availability and genotype. Evidence accumulated over the past decade demonstrates that germination is not a linear process that uniformly increases antioxidant levels. Instead, it represents a dynamic equilibrium in which anabolic synthesis competes with catabolic degradation, often yielding qualitative improvements in solubility, molecular weight distribution and bioaccessibility, even when total phenolic metrics remain unchanged or fluctuate [8,71,72,75,77]. Accordingly, this section examines germination-induced modifications to phenolic acids, flavonoids and tannins and evaluates consequent changes in antioxidant capacity, establishing a framework for optimising sorghum germination parameters to enhance nutraceutical potential.

### 4.2. Effect on Phenolic Enrichment

The enrichment of phenolic compounds during germination reflects a coordinated physiological response to dormancy breakage and the biotic and abiotic stresses associated with early seedling development. This metabolic reconfiguration proceeds primarily through two interrelated pathways: the synthesis of new secondary metabolites and the liberation of phenolics previously immobilised within the grain matrix [72,75].

#### 4.2.1. Activation of the Phenylpropanoid Pathway

De novo phenolic synthesis during germination is driven principally by activation of the phenylpropanoid pathway, with phenylalanine ammonia-lyase (PAL) acting as the rate-limiting enzyme. PAL catalyses the non-oxidative deamination of L-phenylalanine to trans-cinnamic acid, thereby linking primary amino acid metabolism to secondary phenolic biosynthesis [52,76]. Trans-cinnamic acid serves as the precursor for a diverse array of downstream metabolites, including phenolic acids, flavonoids and lignin monomers.

In dormant sorghum seeds, PAL activity is minimal; however, imbibition rapidly induces its expression, reflecting a developmental and defensive strategy whereby the emerging seedlings reinforce cell walls through lignification and produce phytoalexins to counter microbial challenge under warm, moist germination conditions [52,72]. Increased metabolic flux through the phenylpropanoid pathway leads to accumulation of soluble phenolic acids and flavonoids within the radicle, plumule and enzymatically modified endosperm [71,77]. This pathway is further modulated by environmental cues during germination, with factors such as temperature, moisture and light acting as abiotic signals. Light exposure, in particular, has been shown to upregulate flavonoid biosynthetic genes, including chalcone synthase, promoting greater accumulation of anthocyanins and 3-DXAs relative to dark-germinated grains [9,76]. Consequently, the phenolic profile of germinated sorghum represents a plastic phenotype shaped by specific germination conditions.

#### 4.2.2. Enzymatic Hydrolysis and Liberation of Bound Phenolics

Alongside de novo synthesis, liberation of bound phenolics constitutes a nutritionally significant mechanism underpinning germination-induced enrichment. In ungerminated sorghum, a substantial proportion of phenolic acids, particularly ferulic acid, functions as structural cross-linkers between arabinoxylans and lignin within the cell wall [9,74]. These bound forms are largely insoluble and exhibit limited bioaccessibility in the upper gastrointestinal tract.

Germination induces the production of cell wall-degrading enzymes that remodel this architecture. Feruloyl esterases cleave ester linkages between ferulic acid and arabinoxylan side chains, while β-glucosidases hydrolyse glycosidic bonds to release phenolic aglycones from conjugated storage forms, increasing lipophilicity and absorption potential [8,9]. Concurrent activity of xylanases and cellulases further solubilises the polysaccharide framework, enhancing extractability and digestive accessibility of liberated phenolics [72]. This enzymatic remodelling shifts the phenolic profile from one dominated by insoluble, matrix-bound compounds to one enriched in soluble, free forms that display greater antioxidant efficacy in aqueous systems. Release kinetics are typically time-dependent, with maximal liberation observed between 48 and 72 h of germination, coinciding with peak enzyme activity [72,73,75].

#### 4.2.3. The Synthesis–Degradation Equilibrium

**Figure 2:**
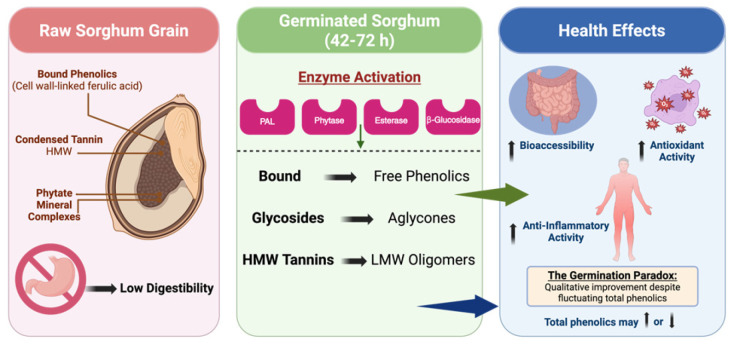
The germination paradox in sorghum polyphenols.

Germination-induced phenolic modulation is governed by a dynamic balance between synthetic and degradative processes. While PAL activation and esterase-mediated hydrolysis expand the pool of available phenolics, oxidative enzymes such as polyphenol oxidase and peroxidase are concurrently induced. These enzymes catalyse oxidation of phenolics to quinones, which may polymerise into insoluble products, including lignin or phlobaphenes [52,76]. This interplay explains the frequently reported “germination paradox”, wherein total phenolic content may stabilise or decline at later stages despite enhanced biological activity (Figure 2). Reductions in bulk phenolic measures often reflect oxidative polymerisation or utilisation of phenolics for structural development, whereas functional gains arise from selective enrichment of low-molecular-weight, highly active species. Thus, the net outcome of germination is not a simple quantitative increase but a qualitative refinement of the sorghum phenolic profile, which balances developmental requirements with enhanced bioactive potential [71,72,75,77].

### 4.3. Impact on Phenolic Acids

Phenolic acids constitute the most abundant class of polyphenols in sorghum, yet in the raw grain they are largely inaccessible, owing to covalent integration within the cell wall matrix. Germination fundamentally alters this availability by shifting the balance from bound storage forms to free, bioactive forms [42,46,72].

#### 4.3.1. Ferulic Acid Dynamics

Sorghum’s predominant phenolic acid, ferulic acid typically accounts for the bulk of total phenolic acid content [42,46]. In raw grain, up to approximately 90% of ferulic acid may be present in bound form, esterified to cell wall arabinoxylans [42,46]. Multiple studies report that germination induces a marked increase in free ferulic acid; during the initial 48 h of germination, enzymatic degradation of the aleurone and endosperm cell walls releases ferulic acid, producing measurable increases in the free fraction [72,73]. The observed dynamics reflect competing processes of release and reutilisation: esterase activity liberates free ferulic acid, whereas oxidative cross-linking associated with the lignification of emerging tissue can consume or reallocate phenolics, such that total ferulic acid (free + bound) may remain constant or decline slightly [72]. From a nutritional standpoint, increases in the free fraction are most relevant because free ferulic acid is available for small intestinal absorption, whereas bound ferulate requires colonic microbial esterases for release [78,79]. Thus, germination effectively “pre-digests” the cell wall, enhancing the bioaccessibility of this antioxidant [72,73].

#### 4.3.2. Other Phenolic Acids

The behaviour of other phenolic acids during germination generally parallels that of ferulic acid, albeit with qualitative differences determined by their structural roles [42,46]. p-Coumaric acid, another principal cell wall constituent often associated with lignin, is likewise released by esterase activity [42]. Metabolomic studies have additionally reported the emergence of simpler phenolic metabolites in germinated flours, such as caffeoylglycerol and protocatechuic acid, which likely derive from partial depolymerisation of complex tannins or hydrolysis of specific ester conjugates absent in the dormant seed [72]. Liberation of gallic acid is notable in tannin-containing varieties: gallic acid, present as galloyl subunits of hydrolyzable tannins or as galloylated esters, can be cleaved during germination, increasing free gallic acid concentrations. Given its pyrogallol moiety, free gallic acid exerts strong radical-scavenging activity, and its release correlates with enhanced antioxidant capacity in aqueous systems [46].

#### 4.3.3. Implications of the Free/Bound Ratio Shift

A hallmark of germination is the pronounced shift in the free:bound phenolic ratio, representing a substantive structural reconfiguration of the grain [72,73]. Where ungerminated sorghum may exhibit a heavily skewed ratio (for example ~20:80 free:bound), germination (e.g., 72 h) often yields a substantially increased free fraction [72]. This remodelling has practical implications for food applications: germinated sorghum flour displays different functional behaviour in food matrices because free phenolics are more mobile and interact more readily with proteins and starches, potentially modifying rheological properties such as dough elasticity and viscosity. More importantly, the greater proportion of free phenolics supplies antioxidants that are immediately available to quench reactive oxygen species in food systems (thereby extending shelf life) and in the upper gastrointestinal tract (reducing oxidative stress) [79,80].

### 4.4. Impact on Flavonoids and 3-DXAs

Sorghum is distinctive among cereals for its elevated flavonoid content, notably the rare 3-DXAs concentrated in the pericarp of red and black varieties [42,46,49]. Germination exerts substantial, genotype-dependent effects on these compounds through both de novo biosynthesis and enhanced extractability [72,73].

#### 4.4.1. De Novo Synthesis of Flavonoids

Consistent with PAL upregulation, the flavonoid biosynthetic pathway is strongly induced during germination [72,73]. Pigmented sorghum varieties frequently demonstrate substantial increases in total flavonoid content; for example, B Li and colleagues reported ~40–50% increases after 48 h of germination [81]. This increase reflects the synthesis of flavones (e.g., luteolin and apigenin) and flavanones (e.g., naringenin) that function as phytoalexins [42]. The temporal kinetics typically follow a sigmoid pattern: a lag phase (0–24 h) with minimal synthesis, an exponential accumulation phase (24–72 h) coinciding with active radicle and coleoptile growth, and a stationary/decline phase (>72 h), during which synthesis plateaus or declines, owing to metabolic utilisation for structural integration or polymerisation into insoluble forms.

#### 4.4.2. Dynamics of 3-DXAs

3-DXAs (e.g., luteolinidin and apigeninidin) are of particular nutraceutical interest due to their exceptional pH and thermal stability and reported bioactivities [46,49]. Germination effects on 3-DXA content vary with genotype and environment: certain red and black genotypes induce 3-DXA synthesis during sprouting, intensifying sprout pigmentation, a process regulated by transcriptional factors, such as Yellow seed1 (y1), which modulate flavonoid pathway flux [82,83]. Conversely, excessive moisture during steeping can cause leaching of water-soluble pigments into steep water, producing an apparent loss. A key distinction emerging from recent work is between absolute concentration and extractability: pericarp matrix degradation by cellulases frequently improves solvent extraction efficiency of 3-DXAs, implying enhanced digestive bioaccessibility even where total synthesis is modest [72]. Additionally, as 3-DXAs can be precursors to phlobaphenes (insoluble reddish/brown polymers), germination conditions favouring oxidation may therefore shift monomeric 3-DXAs into polymeric, insoluble antioxidant fibres, reducing free pigment measurements while increasing insoluble antioxidant content [42,82].

#### 4.4.3. Flavonoid Glycosides Versus Aglycones

In raw sorghum, most flavonoids are present as glycosides (e.g., apigenin-7-O-glucoside) with attached sugar moieties [46,49]. β-Glucosidase activity induced during germination cleaves these glycosidic bonds, releasing aglycones (e.g., apigenin) [73,79]. Aglycones are generally more hydrophobic and have greater capacity for passive diffusion across the intestinal epithelium, rendering this conversion nutritionally favourable [78,79]. Consequently, even when the molar quantity of a flavonoid core remains constant, germination shifts the functional pool toward more bioavailable aglycones. This qualitative transition helps explain observations whereby germinated sorghum flours exhibit augmented anti-inflammatory activity in cellular models despite modest changes in total flavonoid content: the aglycone forms possess greater cellular uptake and efficacy in modulating signalling pathways [19,72,80]. Sorghum exhibits substantial genotype-dependent variation in phenolic/flavonoid composition across global germplasm, and sorghum pigments include both 3-deoxyanthocyanidin aglycones and O-glycosylated derivatives [63,82,84,85,86,87,88]. Notably, this glycoside-to-aglycone conversion is observed across diverse sorghum genotypes (not only pigmented lines), although pigmented sorghums typically show the most pronounced shifts due to their higher baseline flavonoid levels [89,90]. Pigmented sorghums (e.g., tannin or 3-deoxyanthocyanidin-rich types) therefore tend to show the largest absolute compositional differences.

### 4.5. The Tannin Conundrum

Condensed tannins (proanthocyanidins) present a nutritional paradox in sorghum: they confer potent antioxidant activity yet act as anti-nutrients through protein and mineral binding [8,9,42,91]. Germination is widely observed to reduce assayable condensed tannins, though the underlying mechanisms reflect a balance among leaching, enzymatic modification and polymerisation/insolubilisation.

#### 4.5.1. Reduction in Assayable Tannins

Reported reductions in assayable tannins following germination range from ~30% to >60%, contingent on genotype and germination duration [18,71]. Mechanisms accounting for decreased assayability include the following:

1. Leaching: Water-soluble low-molecular-weight tannins may be removed during initial steeping [15].
2. Enzymatic modification: Endogenous polyphenol oxidases (PPO) and peroxidases activated during germination can catalyse oxidative modification or partial degradation of tannin polymers [42,92].
3. Polymerisation and insolubilisation: Tannins may complex with mobilised proteins or cell wall components, forming insoluble complexes that are no longer extractable by standard assay solvents, thereby “disappearing” from analytical windows while remaining in the matrix [42,92].

#### 4.5.2. Shift in Degree of Polymerisation (DP)

Metabolomic profiling indicates that germination shifts tannin profiles from high-molecular-weight (HMW) polymers toward lower-molecular-weight (LMW) oligomers and monomers (e.g., catechin and epicatechin) [9,72,93]. This depolymerisation is nutritionally significant: HMW tannins are primarily responsible for protein precipitation and mineral chelation, whereas LMW oligomers retain radical-scavenging capacity but lack the steric bulk required for efficient protein cross-linking. Consequently, depolymerisation mitigates anti-nutritional effects (improving protein digestibility) while preserving or enhancing antioxidant function [93]. This mechanism explains observations of declining TPC or assayable tannins concurrent with improved biological efficacy following germination [15,18,93].

### 4.6. Evolution of Antioxidant Capacity

The antioxidant capacity of germinated sorghum emerges from the combined compositional changes described above: the liberation of phenolic acids, synthesis of flavonoids and modification of tannins. Antioxidant capacity is multifaceted and assay-dependent; common chemical assays capture differing mechanisms of action [15,56,71,72,94].

#### 4.6.1. Effect on Radical Scavenging Activity

The DPPH and ABTS assays quantify the capacity of compounds to donate electrons or hydrogen atoms to neutralise stable radicals [56,69]. Germination typically enhances DPPH and ABTS scavenging; increased DPPH activity coincides with peak TPC at 48 h [18,56,71]. This enhancement correlates with the liberation of free phenolic acids and synthesis of flavonoids; hydroxyl groups on liberated ferulic acid and synthesised luteolin provide redox centres for radical quenching [8,9]. Improvements are commonly time-dependent, with TEAC (ABTS) values reported to increase progressively up to 72 h in some studies [18,71].

#### 4.6.2. Ferric Reducing Antioxidant Power (FRAP)

FRAP measures the electron-donating capacity of samples by the reduction of Fe^3+^ to Fe^2+^. Germinated sorghum widely exhibits elevated FRAP values [18,71,72,95], indicating that germination-generated phenolics function effectively as electron donors. Such activity is relevant for interrupting oxidative chain reactions in lipids and for chelating pro-oxidant transition metals; high FRAP values in pigmented germinated sorghums are likely driven by conjugated systems in 3-DXAs and tannin-derived oligomers [56].

#### 4.6.3. Oxygen Radical Absorbance Capacity (ORAC)

ORAC assesses chain-breaking antioxidant activity against peroxyl radicals using a fluorescence decay method and is often considered more biologically representative [56,69,94]. Although ORAC data are less consistently reported than DPPH/ABTS, the consensus is that germination increases ORAC values [15,56]. Importantly, the bound phenolic fraction liberated during germination often exhibits high ORAC reactivity; solubilisation of this fraction effectively “activates” potential antioxidant capacity, rendering germinated sorghum flour potentially superior for preventing lipid peroxidation in food systems relative to raw flour [56,72,93].

### 4.7. Genotypic Variations: The Pigmentation Factor

Genetic background, particularly loci governing testa and pericarp pigmentation (B1/B2, R/Y), strongly modulates germination responses [9,42,80].

#### 4.7.1. Pigmented (Red, Brown, and Black) Genotypes

Pigmented sorghums rich in tannins and 3-DXAs generally display the most pronounced responses to germination [9,71]. These varieties possess the following:

- High basal potential: Greater initial phenolic pools for enzymatic mobilisation [9];
- Enzymatic responsiveness: Evidence suggests stronger phenylpropanoid pathway activity, with black sorghum often showing marked increases in FRAP and ABTS post-germination, as tannin reserves are mobilised into lower-molecular-weight antioxidants [18,72];
- Tannin reduction benefits: In tannin-bearing types, germination reduces anti-nutritional tannins and thereby improves protein digestibility, a benefit not applicable to tannin-free white varieties [15,18].

#### 4.7.2. White and Tan Genotypes

White and tan genotypes, lacking condensed tannins and anthocyanins, are dominated by phenolic acids (ferulic and p-coumaric) [9,42]. These varieties typically show the following:

- Moderate quantitative increases: Release of bound phenolic acids raises TPC modestly, though absolute levels remain lower than pigmented types [71,72];
- Functional application: Germination chiefly improves sensory properties (reducing bitterness), lowers phytate, and modifies starch functionality, rendering these genotypes suitable for staple food formulations (porridges and breads) where neutral colour and taste are preferred [15].

### 4.8. Temporal Kinetics: Optimisation of Germination Time

Germination duration is a critical variable; the assumption that “more is better” does not apply indefinitely [15,73,75,81]. Phenolic enrichment follows a defined temporal trajectory requiring optimisation for industrial implementation [18,73,81]:

- 0–24 h (imbibition and activation): Minimal TPC change as enzymes hydrate and activate [15,81].
- 24–48 h (exponential phase): Rapid flavonoid synthesis and phenolic acid release; often the optimal window for maximising TPC and antioxidant capacity [18,71,81].
- 48–72 h (peak and maturation): Phenolics frequently peak; free:bound ratios and bioaccessibility are typically optimal, though rootlet growth increases and some reserves are consumed [18,71,81].
- >72 h (decline and senescence): Phenolics may be utilised for lignification or oxidised by PPO, TPC may plateau or decline, and contamination risks rise [15,75].

For most functional food objectives, a 48–72 h germination window balances phenolic enrichment with practical processing constraints.

### 4.9. Industrial and Nutritional Implications

Germination-driven compositional changes have direct translational relevance. The conversion of bound to free phenolics indicates potential for germinated sorghum flour to act as a natural antioxidant additive to inhibit lipid oxidation in high-fat matrices (baked goods and meats) [8,46,71,81]. A reduction in tannins permits the use of pigmented sorghums, previously limited by astringency in gluten-free and value-added products where colour and antioxidant profile are desirable [8,46,71,81]. The variability in optimal germination durations (48–72 h) enables manufacturers to tailor processes: shorter regimes prioritise antioxidant retention, while longer regimes favour starch modification and sugar generation [18,73,81].

### 4.10. Conclusion

The “germination paradox” resolves into a narrative of qualitative refinement: germination frequently yields improved biological outcomes despite variable or reduced bulk phenolic measures because compositional shifts towards more bioactive, soluble, and lower-molecular-weight species are not captured by aggregate assays [81,96]. A sample may exhibit diminished TPC yet demonstrate stronger cytoprotective effects in cellular models [18,71,72]. These discrepancies highlight the limitations of sole reliance on Folin–Ciocalteu-type assays and the necessity for targeted metabolomic characterisation [72,96,97].

Overall, germination converts sorghum from a repository of bound and polymerised defensive compounds into a delivery system of free, soluble, and metabolically active antioxidants [8,15,18,71,72,81]. Additional research is needed to prioritise the optimisation of germination parameters to maximise desirable bioactive profiles and investigate interactions between germinated sorghum phytochemical profiles and the gut microbiome to bridge chemical potential and physiological bioavailability [72,79,96,97].

## 5. Impact of Germination on Bioaccessibility and Bioavailability of Sorghum Polyphenols

Germination exerts a substantive modulatory effect on the digestive liberation, intestinal absorption, metabolic conversion and systemic distribution of sorghum-derived polyphenols. The sprouting process initiates structural and biochemical transformations that facilitate release and subsequent uptake of phenolic constituents, although the magnitude and direction of these effects depend on germination duration and grain genotype [18,75]. Activation of endogenous hydrolytic enzymes, including β-glucosidases and esterases, cleaves glycosidic linkages and ester bonds, thereby converting polymeric phenolics into smaller, more diffusible forms [15]. Concurrent catabolism of storage macromolecules such as starch and protein remodels the seed matrix, increasing porosity and water accessibility and thus facilitating phenolic mobilisation [18]. In parallel, reductions in anti-nutritional factors, particularly phytic acid and protease inhibitors, attenuate mineral chelation and improve enzymatic digestion [15]. Collectively, these germination-induced alterations in composition and matrix architecture act as a biochemical pre-processing step that shapes the bioaccessibility, absorption and metabolic fate of sorghum polyphenols during gastrointestinal transit.

### 5.1. Bioaccessibility and Bioavailability

The distinction between bioaccessibility and bioavailability represents a fundamental conceptual framework in evaluating the physiological significance of dietary polyphenols [79]. Bioaccessibility denotes the fraction of phenolic compounds that releases during gastrointestinal digestion from the food matrix and rendered soluble in the luminal milieu, thus available for intestinal absorption [98]. Conversely, bioavailability encompasses the subsequent sequence of absorption, phase I/II metabolism, systemic circulation, and tissue distribution, culminating in measurable biological effects [79,98]. In cereals, the disparity between these two parameters is accentuated by the structural localization of phenolics within fibrous or proteinaceous matrices. A limited proportion of aglycone or low-molecular-weight phenolics is absorbed across the small intestine, whereas the majority, existing as insoluble polymers or tannin–fibre complexes, traverse to the colon largely unaltered [79]. Within the large intestine, anaerobic microbial fermentation transforms these bound phenolics into low-molecular-weight derivatives with enhanced permeability and systemic potential. Thus, while bioaccessibility reflects matrix liberation within the gut lumen, bioavailability integrates the comprehensive metabolic and absorptive fate of these compounds. Importantly, improved bioaccessibility through germination constitutes a prerequisite for enhanced systemic bioavailability, even though high release does not necessarily correspond to efficient absorption [18,75].

### 5.2. Germination and Digestive Release of Sorghum Polyphenols

Germination effects a dynamic remodelling of the sorghum grain matrix, which directly influences phenolic release during digestion. Enzymatic hydrolysis mediated by β-glucosidases, esterases and cell wall-degrading hydrolases promotes depolymerisation of conjugated phenolics otherwise trapped within the lignocellulosic network [15]. Metabolomic analyses indicate that early germination (approximately 48–72 h) induces pronounced decomplexation of the matrix, leading to enhanced solubilisation of bound phenolics and increased free:bound ratios [72]. Beyond this interval, the free fraction may decline, reflecting metabolic reassimilation of liberated phenolics by the developing sprout [72]. This temporal pattern implies that germination mediates both the release and turnover of phenolic constituents in a tightly regulated manner.

Qualitatively, germinated sorghum frequently exhibits enrichment of bioactive subclasses, such as flavonols and flavanones [18], even when total phenolic content and classical chemical antioxidant indices display modest or inconsistent changes [71]. These observations indicate that the functional advantage of germination resides less in gross phenolic accumulation and more in the generation of smaller, more redox-active compounds with improved solubility and cellular penetrability. Concurrent reductions in tannins and phytic acid further augment the accessibility of low-molecular-weight phenolics by alleviating steric and chemical constraints on their release [15,18]. Within this framework, germination periods of 48–72 h typically maximise the liberation of bioactive phenolics prior to substantial reutilisation or degradation by the sprout [15,18,72].

Standardised simulated gastrointestinal digestion models corroborate the enhancing effect of germination on polyphenol bioaccessibility. INFOGEST in vitro assays applied to germinated sorghum report elevated phenolic recovery, particularly during the oral and early gastric phases, attributable to matrix softening and partial depolymerisation of cell wall components [72]. As digesta advances through gastric and intestinal phases, some flavonoids decline, owing to acid or enzymatic degradation; however, digestion and microbial metabolism can generate smaller phenolic metabolites that may remain bioaccessible and contribute to antioxidant potential [79,81,99]. For example, in vitro digestion of black sorghum produced a two-fold increase in total phenolic concentration and a four-fold enhancement of kaempferol-3-O-xyloside post-digestion [99]. Pre-germination likely potentiates such transformations by weakening matrix barriers and improving enzyme access to phenolic substrates. Dynamic digestion systems that emulate peristalsis and secretions often report higher phenolic and antioxidant recoveries than static models, suggesting that conventional assays may underestimate physiological bioaccessibility [79]. Collectively, these data support germination as a critical bioprocess that enhances the digestive liberation and solubility of sorghum polyphenols, thereby expanding the pool of compounds available for subsequent absorption and metabolism.

### 5.3. Transepithelial Transport and Phase II Metabolism

Following release from the sorghum matrix, polyphenols must traverse the intestinal epithelium and undergo substantial metabolism before producing systemic effects. The Caco-2 cell monolayer differentiating into enterocyte-like cells with tight junctions and brush border microvilli remains the principal in vitro model for delineating transepithelial transport mechanisms [79]. Application of digested sorghum extracts to Caco-2 monolayers has revealed the emergence of multiple transported phenolic derivatives, including trans-pinostilbene and maackin A, indicating that cellular uptake is closely linked to biotransformation processes intrinsic to intestinal metabolism [99]. Comparable patterns are reported for germinated quinoa, where in vitro digestion/Caco-2 models demonstrate that germination alters both the profile and abundance of transported metabolites [100].

These observations underpin the so-called “bioavailability paradox”: apical transport of parent compounds is often limited (for example, ~10–15% for ferulic acid), yet when intestinal and hepatic compartments are integrated in Caco-2/HepG2 co-culture systems, downstream metabolites increase substantially (160–370%), reflecting extensive phase II biotransformation despite modest parent compound flux [79,101]. Human intervention studies with pigmented cereals similarly report low proportions of circulating parent phenolics (<6%) alongside significant improvements in plasma antioxidant capacity [98,102]. Accordingly, the physiologically relevant metric of “functional bioavailability” resides not in the parent phenolic fraction but in the composite pool of metabolites generated post-absorption and hepatic transformation [98,102].

Direct comparisons between germinated and ungerminated sorghum in transport models are limited, but the germination-induced shift toward low-molecular-weight, more soluble phenolics and enhanced initial bioaccessibility would be expected to enlarge the substrate range available for transepithelial transport and conjugation. Phase I/II reactions within enterocytes and hepatocytes including glucuronidation, sulfation and O-methylation, yield conjugated derivatives such as luteolin glucuronide that circulate at low micromolar concentrations yet retain biological activity. Evidence from germinated legumes demonstrates the physiological relevance of such derivatives: polyphenols from germinated beans enriched in vitexin and isovitexin improved glycaemic regulation in diabetic mice, indicating that germination-derived metabolites can exert systemic effects beyond those of parent compounds [48,103]. In summary, germination influences absorption and metabolism primarily by increasing the pool and diversity of bioaccessible sorghum phenolics available for transepithelial transport and metabolic conjugation.

### 5.4. Gut Microbiota and Colonic Transformation

A substantial portion of sorghum polyphenols, particularly high-molecular-weight tannins and complex flavonoids, escape small intestinal absorption and reach the colon, where they undergo extensive microbial catabolism. Colonic bacteria expressing esterases, reductases and glycosidases depolymerise sorghum tannins and other complex phenolics into phenylpropionic and benzoic acid derivatives that are readily absorbed and further conjugated to hippuric acid, a recognised urinary biomarker of dietary polyphenol exposure [101]. Sorghum-specific 3-DXAs are likewise degraded to low-molecular-weight phenolic acids detectable in human plasma [56,104]. These microbial conversions thus constitute an integral extension of polyphenol metabolism, generating small metabolites that contribute to systemic antioxidant and anti-inflammatory effects.

The host–microbe interaction is reciprocal. Phenolics derived from sorghum bran exert prebiotic effects, modulating microbial ecology by stimulating Roseburia, Prevotella, Bifidobacterium, and Lactobacillus populations, which in turn support epithelial barrier function and short-chain fatty acid production [79,105]. Germination augments the pool of bioaccessible substrates entering the colon by increasing release of phenolics in the upper gut and by shifting their molecular profile toward more soluble and partially depolymerised forms, thereby amplifying the scale and nature of microbial transformations and ecological shifts [18,72]. Ongoing clinical investigations, including a 2025 trial in obese adults testing phenolic-rich “sumac” sorghum for gut microbiota and inflammatory modulation, reflect growing translational interest in the germinated sorghum–microbiota axis.

Overall, germination affects sorghum polyphenols at multiple hierarchical levels: it enhances digestive release and host absorption and expands the repertoire of substrates available for colonic fermentation and microbial–host co-metabolism. The resulting network of conjugated and microbially derived metabolites provides a plausible mechanistic basis for the local and systemic health effects attributed to germinated sorghum [79,98].

### 5.5. Comparative Insights from Other Grains and Legumes

Germination-induced remodelling of sorghum polyphenols occurs within a broader context in which sprouting modulates phenolic profiles and functionality across cereals and legumes. Across matrices, germination typically produces a characteristic pattern: qualitative shifts from high- to low-molecular-weight phenolics, generally improved bioaccessibility and antioxidant potential, and heterogeneous trends in total phenolic content (TPC), which may increase, plateau or decline depending on the genotype and processing conditions. Sorghum conforms to this general paradigm but is distinctive in the prominence of high-molecular-weight condensed tannins that are converted into smaller, more bioavailable phenolics during germination [71,72].

In cereals, malting and sprouting consistently enhance subfractions of the phenolic spectrum in a class-specific manner. Barley germination selectively increases bioaccessible flavones, while flavan-3-ols and anthocyanins are comparatively refractory to enzymatic liberation [22,102]. Wheat sprouting augments flavonoids, phenolic acids and benzoxazinoids, and germinated brown rice exhibits increased concentrations of free phenolics, together with other bioactive constituents such as p-coumaric acid and γ-aminobutyric acid. These cereal examples parallel sorghum in that germination tends to favour the liberation of more soluble, lower-molecular-weight compounds and to improve functional properties, even though TPC responses differ among matrices and phenolic subclasses.

In legumes, germination exerts a dual effect on nutritional and phytochemical quality by enhancing protein quality alongside phenolic abundance [15,28]. Mung bean germination, for example, increases TPC approximately 3.07-fold and enriches vitexin and isovitexin, changes that coincide with hypoglycaemic activity in in vivo model [103]. Conversely, chickpea sprouting for 48 h increased total phenolics by ~35% while reducing bioaccessibility by a similar magnitude, suggesting that newly synthesised or rearranged compounds may display lower stability or stronger matrix binding [26,35,106]. Such nonlinearity echoes sorghum findings in which qualitative transformations, particularly depolymerisation of high-molecular-weight tannins to smaller, more bioavailable phenolics, can outweigh gross TPC changes as determinants of functional performance [71,72].

Collectively, comparative evidence from barley, wheat, rice and pulses supports the interpretation that germination functions as a broadly applicable bioenhancing strategy across plant matrices, promoting shifts towards more accessible and functionally active phenolic profiles. Sorghum aligns with this paradigm while simultaneously highlighting key limitations: increases in TPC are neither necessary nor sufficient for improved bioactivity, and the most consequential changes frequently involve qualitative restructuring of the phenolic pool, a theme particularly salient for tannin-rich cereals and legumes.

### 5.6. Key Caveats and Considerations

Despite the substantial enhancement of phenolic liberation and bioaccessibility achieved through germination, several experimental and physiological caveats must be acknowledged in interpreting these findings. The duration of germination continues to be a crucial determinant of phenolic yield; extension of sprouting beyond approximately seventy-two hours frequently leads to metabolic re-assimilation of liberated phenolics by the developing embryo, thereby diminishing extractable content [72]. Furthermore, enzymatic oxidation during late germinative stages may degrade thermolabile phenolics, producing an apparent decline in total phenolic content despite concurrent reductions in anti-nutritional factors such as tannins and phytates [15]. This phenomenon accentuates the complex trade-off between anti-nutrient attenuation and phenolic stability.

Inter-individual variability further complicates the extrapolation of in vitro results to human populations. Differences in gut microbial composition, enzymatic activity, and phase II conjugation capacity contribute to marked heterogeneity in absorption kinetics and metabolic profiles [102]. Consequently, identical dietary exposures may yield divergent metabolite spectra and biological outcomes among individuals. Moreover, while in vitro systems such as Caco-2 monolayers and standardised INFOGEST digestion models provide valuable mechanistic insight, they represent simplified approximations of the human gastrointestinal environment. These models may underestimate or exaggerate polyphenol release, stability, and transepithelial transport, highlighting the necessity for corroboration through controlled human interventions and metabolomic profiling.

An additional conceptual consideration pertains to the distinction between local and systemic efficacy. Even when circulating concentrations of parent or conjugated phenolics remain low, substantial physiological benefits may arise from luminal and mucosal interactions. Polyphenols and their metabolites can exert antioxidant, anti-inflammatory, and microbiota-modulatory effects directly within the intestinal milieu, enhancing epithelial barrier integrity and influencing short-chain fatty acid production. Hence, the functional value of germinated sorghum extends beyond measurable systemic uptake to include localised intestinal mechanisms that contribute to overall metabolic and immune homeostasis.

In summary, germination fundamentally restructures the sorghum grain matrix, promoting enzymatic liberation and transformation of phenolic compounds into a metabolically active pool that participates in both host and microbial antioxidant networks. Through the coordinated processes of enzymatic hydrolysis, microbial bioconversion, and phase II conjugation, these metabolites collectively confer local and systemic protective effects, encompassing redox balance, inflammatory regulation, and vascular health. The optimisation of germination parameters, integration with dynamic digestion–absorption models, and inclusion of inter-individual microbiome analyses constitute essential directions for future investigations into sorghum’s nutraceutical potential.

## 6. Bioactivity of Germinated Sorghum Polyphenols: Evidence from In Vitro, In Vivo and Human Studies

Germination modifies the bioactivity of sorghum polyphenols in ways that are not fully captured by compositional metrics or simple chemical antioxidant assays. Across in vitro, in vivo and human intervention models, a recurring pattern emerges in which germination may reduce bulk extractable antioxidant capacity, as measured by assays such as DPPH or FRAP, yet enhance biologically relevant endpoints, including cellular antioxidant protection, metabolic regulation and anti-inflammatory efficacy [18,71,72,81]. This apparent discrepancy reflects a qualitative reconfiguration of the phenolic profile from a state dominated by bound, high-molecular-weight tannins and matrix-sequestered phenolic acids to one enriched in more soluble, lower-molecular-weight species and aglycones with superior cellular uptake and signalling capacity [8,9].

### 6.1. In Vitro and Ex Vivo Cellular Bioactivity

In vitro investigations comparing raw and germinated sorghum consistently demonstrate that germination can diminish total phenolic content and reduce radical scavenging capacity in purely chemical systems, while at the same time enhancing antioxidant performance in cell-based assays [71,72,81]. When germinated sorghum extracts are applied to erythrocyte or other cellular models subjected to oxidative insult, they frequently provide greater protection against membrane lipid peroxidation and haemolysis than extracts from ungerminated grain, despite lower DPPH or FRAP values [71]. This pattern indicates that germination generates phenolic profiles with improved membrane partitioning and intracellular accessibility, favouring phenolic acids and flavonoid aglycones that are more effective at intercepting peroxyl radicals in lipid bilayers and at modulating redox-sensitive signalling pathways [8,9]. Parallel work on enzyme inhibition further brings attention to the qualitative shift: while germination tends to reduce angiotensin-converting enzyme (ACE) inhibitory activity, likely through proteolytic degradation of specific peptide motifs, it can preserve or enhance the inhibition of α-amylase and α-glucosidase via smaller flavonoids and phenolic acids that interact more selectively with enzyme active sites [107,108]. Collectively, these findings support the view that germination refines sorghum’s in vitro bioactivity from predominantly non-specific, tannin-driven effects towards more targeted and physiologically compatible mechanisms [18,71,72].

### 6.2. In Vivo Metabolic Regulation and Bioactivity

In vivo studies in rodent models provide convergent evidence that germinated or otherwise bioprocessed sorghum exerts antidiabetic, antioxidant and organ-protective effects that extend beyond those predicted from in vitro radical scavenging [81,109,110]. In chemically induced or diet-induced diabetic mice, sorghum seed extracts derived from germinated or sprouted material have been associated with marked reductions in fasting glycaemia, prevention of diabetes-related weight loss and improvements in glucose tolerance that approach or, in some cases, rival those of reference hypoglycaemic agents at the doses tested [29,111,112,113]. These glycaemic benefits appear to arise from multiple mechanisms, including the inhibition of intestinal carbohydrate-hydrolysing enzymes, suppression of hepatic gluconeogenesis and enhancement of peripheral insulin sensitivity via upregulation of nuclear receptors such as PPAR-γ. At the same time, germinated sorghum interventions repeatedly ameliorate oxidative stress, lowering tissue malondialdehyde concentrations and restoring endogenous antioxidant enzyme activities, including superoxide dismutase and glutathione peroxidase [109,110,114]. These redox adaptations are compatible with the activation of Nrf2-dependent cytoprotective pathways and phase II enzyme induction, consistent with the capacity of sorghum 3-DXAs to engage antioxidant response element signalling [11,110]. Inflammatory outcomes are similarly modulated, with reductions in pro-inflammatory cytokines, attenuation of tissue damage, and evidence of suppressed NF-κB signalling in models of metabolic and inflammatory stress [109,110]. In several studies, these systemic effects co-occur with alterations in gut microbial activity and short-chain fatty acid production, implicating indirect actions along the gut–liver–adipose axis [105,110].

### 6.3. Human Clinical Evidence: Acute Metabolic and Satiety Responses

Human data on germinated sorghum remain limited but provide important proof of concept that germination-induced changes in polyphenol bioactivity translate into measurable physiological effects. Acute, randomised, crossover trials using beverages formulated with germinated and extruded sorghum have shown that such products can modulate postprandial metabolism in normoglycaemic adults [113]. Consumption of germinated sorghum beverages has been associated with enhanced secretion of glucagon-like peptide-1 (GLP-1), an incretin hormone that augments glucose-dependent insulin release and suppresses glucagon, while simultaneously reducing or maintaining the postprandial insulin response required to manage a given glycaemic load [113]. This “insulin-sparing” profile suggests improved efficiency of glucose handling and aligns with the insulin-sensitising and gut-mediated mechanisms observed in animal studies [114,115]. In addition, participants report greater subjective fullness and reduced hunger after ingesting germinated sorghum beverages compared with control formulations, implying that modifications to fibre structure, residual tannin content and polyphenol composition during germination can act in concert with incretin signalling to enhance satiety [113]. Although these clinical trials are short-term and focus on acute responses, they establish a translational link between germination-driven polyphenol remodelling and beneficial effects on metabolic and appetite-related endpoints in humans.

### 6.4. Integrated Mechanistic Interpretation: Reconciling Composition and Function

When considered together, in vitro, in vivo and human data converge on a mechanistic model in which germination acts as a bioavailability gatekeeper for sorghum polyphenols. By activating endogenous esterases, glycosidases and cell wall-degrading enzymes, germination reduces the pool of bound and high-molecular-weight tannins and increases the fraction of free phenolic acids, flavonoid aglycones and low-molecular-weight oligomers capable of crossing biological membranes and engaging intracellular targets [18,72,81]. This reconfiguration explains why chemical antioxidant capacity and total phenolic measures may decline while functional bioactivity improves: the phenolic profile becomes less abundant but more potent per unit absorbed, favouring signalling interactions such as Nrf2 activation, NF-κB inhibition, PPAR-γ modulation and incretin stimulation [11,115]. At the same time, partial depolymerisation and structural modification of tannins and other macromolecular complexes reduce anti-nutritional effects and broaden the prebiotic spectrum available to the gut microbiota, further amplifying systemic benefits through enhanced short-chain fatty acid production and gut barrier support [97,105,114]. However, the existing evidence base remains constrained by heterogeneity in germination protocols, limited genotype coverage and the scarcity of long-term human intervention studies [18,71,72,81]. Within these constraints, current in vitro, in vivo and acute clinical findings nonetheless suggest that germination transforms sorghum from a chemically antioxidant but poorly accessible grain into a more biologically active functional ingredient capable of modulating oxidative stress, inflammation and metabolic regulation along multiple interconnected pathways [8,9,115].

## 7. Summary and Future Perspectives

Germination constitutes a critical biotransformation phase in the post-harvest development of *Sorghum bicolor*, wherein the orchestrated activation of endogenous hydrolytic enzymes profoundly remodels the biochemical, structural, and functional integrity of the grain. Through the concerted action of α-amylases, proteases, β-glucosidases and phytases, the dormant endosperm undergoes progressive degradation of its complex starch–protein matrix, thereby enhancing nutrient digestibility and bioaccessibility. The hydrolytic cleavage of esterified and glycosidic bonds concomitantly dismantles anti-nutritional constituents such as phytic acid, which otherwise chelates divalent cations and impedes phenolic absorption [15]. Moreover, germination induces de novo synthesis of secondary metabolites via the upregulation of key phenylpropanoid pathway enzymes, particularly phenylalanine ammonia-lyase, resulting in an amplified accumulation of low-molecular-weight phenolic acids and flavonoids [10]. Collectively, these processes shift the grain from a metabolically quiescent to a biochemically dynamic state, characterised by enhanced antioxidant potential and improved physiological relevance.

The biochemical enrichment observed during germination is consistently associated with a marked elevation in total extractable phenolic content, often exceeding 40–50% within 72 h, and a parallel two-fold increase in in vitro radical-scavenging activity. In pigmented sorghum varieties, this enhancement is particularly notable for the 3-DXAs apigeninidin and luteolinidin, pigments unique to the species that contribute both chromatic intensity and potent antioxidative capacity [49]. Mechanistically, these compounds exert their protective effects through direct radical scavenging, metal chelation, and the suppression of oxidative enzyme systems. Studies using murine macrophage models (RAW 264.7) have further demonstrated that germinated sorghum extracts attenuate nitric oxide synthesis via downregulation of inducible nitric oxide synthase (iNOS) expression, implicating inhibition of the nuclear factor κB (NF-κB) signalling pathway as a central anti-inflammatory mechanism [8]. This suppression of NF-κB activity subsequently reduces VDthe release of pro-inflammatory cytokines, like tumour necrosis factor-α (TNF-α), interleukin-6 (IL-6) and interleukin-1β (IL-1β), indicating that germination augments the immunomodulatory properties inherent to sorghum polyphenols.

The improved functional efficacy of germinated sorghum has been attributed not only to its elevated phenolic concentration but also to the increased bioaccessibility of these metabolites. The enzymatic loosening of the grain matrix facilitates solubilisation and intestinal release of phenolic compounds, enhancing their potential bioavailability. Simulated gastrointestinal digestion and Caco-2 cell transport models substantiate this observation, revealing that the bioaccessible fraction of total phenolics can increase from approximately 67% in raw grains to as high as 119% post-germination, suggesting the liberation of previously insoluble bound forms [72,81,99]. Nevertheless, the translational interpretation of these data remains constrained by the scarcity of controlled human studies. The majority of evidence derives from in vitro or animal models, and considerable heterogeneity in germination protocols relating to duration, temperature and moisture regime, together with genotypic variation in enzymatic activity and phenolic profiles, limits inter-study comparability [10,13].

Future investigations must therefore be directed toward bridging this translational divide through rigorously designed human intervention trials. Randomised controlled studies are required to determine whether dietary intake of germinated sorghum produces quantifiable modulation of systemic oxidative stress biomarkers such as plasma F2-isoprostanes, total antioxidant capacity, and circulating inflammatory mediators, including C-reactive protein and cytokine panels. Complementary multi-omics approaches integrating metabolomics and transcriptomics will be essential to elucidate the molecular pathways underpinning germination-induced metabolic reprogramming and to delineate the absorption, distribution, metabolism and excretion (ADME) profiles of the principal phenolic constituents. Particular attention should be directed toward the interaction between germination-derived phytochemicals and the gut microbiome, as microbial catabolism of 3-DXAs and complex polyphenols into low-molecular-weight derivatives likely represents a decisive determinant of bioefficacy [8].

Parallel research efforts must also target agronomic and technological optimisation to enhance the translational viability of germinated sorghum as a functional food ingredient. Standardisation of genotype-specific germination protocols, possibly through the inclusion of abiotic elicitors, such as ultraviolet irradiation or controlled osmotic stress, could maximise the yield of desired bioactive compounds [10,116]. Integration of these biochemical insights into breeding programmes should prioritise cultivars exhibiting both elevated basal phenolic content and enhanced enzymatic responsiveness during germination. Furthermore, addressing technological barriers such as the reduced shelf-stability and altered sensory attributes of sprouted flours remains imperative. Stabilisation strategies including kiln drying, enzymatic deactivation or post-germination fermentation [15] may mitigate these constraints. Additionally, the extent to which germination-enhanced antioxidant properties are retained through subsequent processing (e.g., baking and extrusion) warrants attention. Heat and other processing steps can partially degrade polyphenols, so future research should explore post-germination processing methods that preserve these antioxidant gains in final food products. The convergence of biochemical innovation, clinical validation, and sustainable processing technologies thus positions germinated sorghum as a promising platform for the development of next-generation functional foods and nutraceuticals targeting oxidative stress and inflammation-mediated chronic disease pathways [117].

## Figures and Tables

**Table 1:** Fate of germination across cereals and legumes.

Crop	Germination Conditions	Functional Assays/Model	Key Metabolic & Bioactive Outcomes
Sorghum (red/white) [28]	37 °C, 69 h, 80–90% relative humidity; whole grains then extruded at 137 °C.	Chemical: total phenolic content (TPC), ABTS, DPPH. Enzymatic: α-amylase and α-glucosidase inhibition.	TPC increased by ~26%; antioxidant activity increased by ~97%. Strong hypoglycaemic potential (IC50) via digestive enzyme inhibition. Extrusion preserved phenolics and increased soluble fibre.
Sorghum [29]	Ethanolic extract (80% MeOH) incorporated into diet at 0.5% and 1% (*w*/*w*).	Animal: high-fat diet mice. Assays: serum glucose and insulin; adipose tissue Western blots for PPAR-γ, adiponectin, TNF-α.	1% sorghum extract significantly reduced serum insulin and glucose. Upregulated PPAR-γ and adiponectin; downregulated TNF-α, improving insulin sensitivity.
Sorghum sprouts [30]	Sorghum sprout flour produced by germination, then irradiated with UV-A LED light.	Cell: RAW 264.7 macrophages. Assays: in vitro digestion recovery, NO production, antioxidant properties; sensory evaluation of granola bars.	UV irradiation increased gallic acid; sprouting increased catechin. Digested extracts significantly reduced NO production. Granola bars showed good sensory acceptance (≈5.5–6.5/9).
Wheat (Triticum aestivum) [31]	Germination for 1–7 days at 12–21 °C; optimal anti-inflammatory profile at 21 °C for 7 days.	Cell: LPS-induced RAW 264.7 macrophages. Chemical: HPLC–MS profiling of soluble phenolic acids.	Anti-inflammatory activity (TNF-α inhibition) peaked at 21 °C for 7 days. Strong linear correlation between soluble phenolics and TNF-α inhibition. Mobilisation of ferulic acid derivatives from bound to soluble forms
Germinated brown rice [32]	Brown rice germinated with bran/germ retained; diet containing 60% germinated brown rice vs. white rice.	Animal: LDL receptor-knockout (LDLr-KO) mice. Ex vivo: monocyte adhesion to aorta; vascular inflammation markers.	Germinated brown rice reduced atherosclerosis severity. No major change in plasma cholesterol, but reduced vascular inflammation (ICAM-1, MCP-1). Reduced monocyte adhesion to vascular endothelium.
Germinated oats (Avena sativa) [33]	Commercial germinated oat products screened; germination optimised to maximise phytochemicals.	Cell: RAW 264.7 macrophages (NO inhibition). Animal: DSS-induced colitis mice.	Germination increased avenanthramides and other phytochemicals. Germinated oat extract strongly inhibited NO and reduced colitis severity. Gut protection linked to enriched avenanthramides.
Germinated barley foodstuff (GBD) [34]	Germinated barley foodstuff (GBF) prepared from germinated barley fractions rich in hemicellulose fibre.	Animal: DSS-induced colitis mice.	GBF reduced epithelial inflammatory response. Inhibited NF-κB binding and STAT3 expression. Increased caecal butyrate, acting as a prebiotic to improve colonic environment.
Chickpea [35]	Chickpea sprouted for 2 days; diet containing 20% germinated seeds.	Animal: ovariectomised (OVX) rats as an oestrogen-deficiency model. Assays: serum lipid profile and organ weights (including uterus).	Normalised lipid profile (↓ TC, TG, LDL; ↑ HDL). Prevented uterine atrophy, indicating strong phytoestrogenic effects. Lipid-lowering efficacy broadly comparable to atorvastatin in this model.
Cowpea (Bombay) [36]	Cowpea seeds soaked overnight and germinated for 2 days; test diets with 20% sprouted cowpea powder (plus boiled and raw variants).	Animal: high-fat diet rats. Assays: caecal fermentation indices, lipid profile.	Boiled, sprouted and raw cowpea diets modulated high-fat-diet-induced hypercholesterolaemia. Increased caecal Lactobacillus population and caecal weight. Improved serum cholesterol via fibre- and microbiota-mediated mechanisms.
Lentil sprouts [37]	Lentils germinated for 6 days to produce melatonin-rich sprouts.	Animal: Sprague Dawley rats. Assays: plasma melatonin pharmacokinetics; FRAP and ORAC antioxidant capacity.	Germination substantially increased lentil melatonin content. Sprout intake led to rapid melatonin absorption (Tmax ≈ 90 min). Significant increases in plasma antioxidant capacity (FRAP, ORAC).
Mung bean [38]	Anaerobic germination and fermentation of mung beans; aqueous extracts from untreated, germinated and fermented seeds.	Cell: RAW 264.7 macrophages (NO inhibition). Animal: mice ear oedema and hot-plate pain models.	Germinated and fermented mung bean extracts showed potent NO inhibition in vitro. At higher doses, significantly reduced arachidonic acid-induced ear oedema. Strong antinociceptive (analgesic) responses in hot-plate tests.
Quinoa (red/yellow) [39]	Red and yellow quinoa seeds germinated at 17 °C, 90% RH for up to 6 days; 6-day sprouts extracted in ethanol.	Animal: CCl_4_-induced oxidative stress rats.	Germination increased phenolics, flavonoids and carotenoids; red sprouts richer than yellow. Sprout extracts (30 mg GAE/kg) reduced ALT, AST, bilirubin and LDL/VLDL, and increased HDL. Improved antioxidant status (↑ GSH, SOD; ↓ MDA) and showed strong hepatoprotective effects.
Millet (finger/pearl) [40]	Millet (Pennisetum glaucum) germinated at 30 °C for 24 h; flour incorporated into a high-fat, high-fructose diet.	Animal: rats fed a high-fat, high-fructose diet. Assays: intestinal permeability, SCFA profile, goblet cell histology, gut microbiota.	Germinated millet flour improved intestinal health by reducing permeability. Increased caecal propionate (SCFA) and goblet cell number. Beneficially modulated gut microbiota composition (including higher Eggerthellaceae).

The solid upward arrow indicates the elevation while the downward arrow signifies the attenuation.

## Data Availability

No new data were created or analysed in this study. Date sharing is not applicable to this article.

## Abbreviations

The following abbreviations are used in this manuscript:

PPAR-γ	Peroxisome Proliferator-Activated Receptor Gamma
ICAM-1	Intercellular Adhesion Molecule-1
STAT-3	Signal Transducer and Activator of Transcription 3
MCP-1	Monocyte Chemoattractant Protein-1
MeOH	Methanol
DPPH	2,2-Diphenyl-1-picrylhydrazyl
ABTS	2,2′-Azino-bis(3-ethylbenzothiazoline-6-sulfonic acid
TNF-α	Tumour Necrosis Factor Alpha
NF-κB	Nuclear Factor Kappa-Light-Chain-Enhancer of Activated B Cells
SCFA	Short-Chain Fatty Acid
FRAP	Ferric Reducing Antioxidant Power
ORAC	Oxygen Radical Absorbance Capacity
CCl_4_	Carbon Tetrachloride
GSH	Reduced Glutathione
SOD	Superoxide Dismutase
MDA	Malondialdehyde
HDL	High Density Lipoprotein
AST	Aspartate Aminotransferase
ALT	Alanine Aminotransferase
TPC	Total Phenolic Content
LPS	Lipopolysaccharide
DSS	Dextran Sodium Sulphate
TG	Triglycerides
TC	Total Cholesterol
NO	Nitric Oxide
